# Precision Medicine in Cardiovascular Disease Prevention: Clinical Validation of Multi-Ancestry Polygenic Risk Scores in a U.S. Cohort

**DOI:** 10.3390/nu17050926

**Published:** 2025-03-06

**Authors:** Małgorzata Ponikowska, Paolo Di Domenico, Alessandro Bolli, George Bartholomew Busby, Emma Perez, Giordano Bottà

**Affiliations:** 1 Allelica Inc., San Francisco, CA 94105, USA; malgorzata.ponikowska@gumed.edu.pl (M.P.); alessandro@allelica.com (A.B.);; 2 Department of Biology and Medical Genetics, Faculty of Medicine, Medical University of Gdansk, 80-210 Gdansk, Poland; 3 Brigham and Women’s Hospital, Boston, MA 02115, USA

**Keywords:** polygenic risk score, ancestry-specific polygenic risk score, coronary artery disease, coronary artery disease polygenic risk score, PRS, CAD, CAD PRS, precision cardiovascular care

## Abstract

Background: Polygenic risk score (PRS) quantifies the cumulative effects of common genetic variants across the genome, including both coding and non-coding regions, to predict the risk of developing common diseases. In cardiovascular medicine, PRS enhances risk stratification beyond traditional clinical risk factors, offering a precision medicine approach to coronary artery disease (CAD) prevention. This study evaluates the predictive performance of a multi-ancestry PRS framework for cardiovascular risk assessment using the All of Us (AoU) short-read whole-genome sequencing dataset comprising over 225,000 participants. Methods: We developed PRSs for lipid traits (LDL-C, HDL-C, triglycerides) and cardiometabolic conditions (type 2 diabetes, hypertension, atrial fibrillation) and constructed two metaPRSs: one integrating lipid and cardiometabolic PRSs (risk factor metaPRS) and another incorporating CAD PRSs in addition to these risk factors (risk factor + CAD metaPRS). Predictive performance was evaluated separately for each trait-specific PRS and for both metaPRSs to assess their effectiveness in CAD risk prediction across diverse ancestries. Model predictive performance, including calibration, was assessed separately for each ancestry group, ensuring that all metrics were ancestry-specific and that PRSs remain generalizable across diverse populations Results: PRSs for lipids and cardiometabolic conditions demonstrated strong predictive performance across ancestries. The risk factors metaPRS predicted CAD risk across multiple ancestries. The addition of a CAD-specific PRS to the risk factors metaPRS improved predictive performance, highlighting a genetic component in CAD etiopathology that is not fully captured by traditional risk factors, whether clinically measured or genetically inferred. Model calibration and validation across ancestries confirmed the broad applicability of PRS-based approaches in multi-ethnic populations. Conclusion: PRS-based risk stratification provides a reliable, ancestry-inclusive framework for personalized cardiovascular disease prevention, enabling better targeted interventions such as pharmacological therapy and lifestyle modifications. By incorporating genetic information from both coding and non-coding regions, PRSs refine risk prediction across diverse populations, advancing the integration of genomics into precision medicine for common diseases

## 1. Introduction

Atherosclerotic Cardiovascular Disease (ASCVD) is an umbrella term for diseases caused by the build-up of atherosclerotic plaques irrespective of their location in the vascular system. Coronary Artery Disease (CAD), also known as Coronary Heart Disease (CHD), is a condition in which narrowing of the coronary arteries results in an insufficient supply of blood and consequently oxygen to the myocardium. The main cause of CAD is atherosclerosis [1]. In 2022 the worldwide age-standardized prevalence of CAD was 3.6% although there is a high geographical variation [2]. Different risk models have been developed to estimate 10-year risk of ASCVD in various populations. These models help determine when intervention, such as statin initiation, is warranted. For example, the Systematic Coronary Risk Evaluation (SCORE) assesses the 10-year risk of fatal ASCVD in European populations; the Pooled Cohort Equations (PCE) are recommended to be used to estimate 10-year risk of ASCVD in the United States [3]; and China-Par predicts 10-year risk of ASCVD in the Chinese population [4]. The models must be developed using data derived from the same population in which they will be applied. This necessity arises from evidence demonstrating that ancestry influences the accuracy of a model, e.g., for identical calculated PCE values, individuals of South Asian ancestry exhibit a two-fold higher risk of CAD compared to those of European ancestry [5]. Therefore, although the risk factors used are similar in different models, their weighting varies depending on the population used to develop the model.

**Figure 1:**
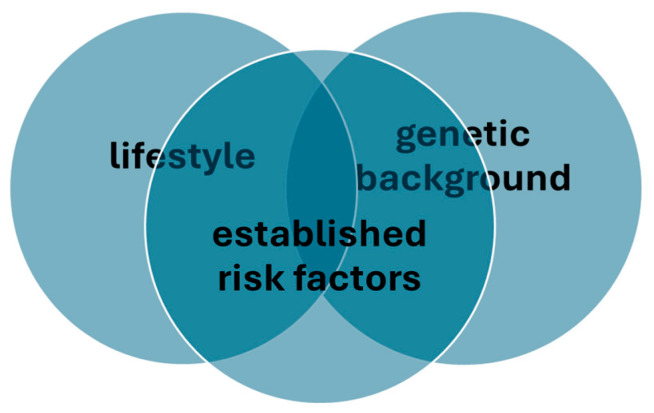
Risk factors of coronary artery disease (CAD). Among the established risk factors of CAD are those dependent on lifestyle, the genetic background or both, as well as those belonging to either category (e.g., age). The proportion of the circles is not representative of the weight of contributions.

The incorporated risk factors in the models include those that can be modified through dietary, lifestyle, and therapeutic interventions, and those that cannot, such as age, sex, and genetic ancestry (Figure 1). Whilst non-modifiable risk factors potentially contribute more than 50% of the predictive performance of CAD risk models [6], there is proven clinical evidence supporting the efficacy of controlling modifiable risk factors, emphasizing their importance in both the development and, therefore, prevention of ASCVD. Modifiable risk factors include hypertension (HT), hyperlipidemia, diabetes mellitus (DM), obesity, smoking, poor diet, and sedentary lifestyle [7]. Although these factors can be modified through external intervention, many of them are also directly or indirectly influenced by the genetic makeup of a patient [8]. However, the genetic component to most of the ASCVD risk factors is rarely the result of established causal, monogenic variants. For example, whilst clinically definite or probable diagnosis of familial hypercholesterolemia (FH) causing extreme levels of LDL-cholesterol (LDL-C) is present in 2.3% of patients with stable CAD [9], only 15–50% of them are found to have an explanatory pathogenic variant [10], meaning that other genetic risk factors are likely playing a role.

CAD itself has an estimated heritability of 40–60% [3], confirming that disease development requires a combination of genetic risk, as well as certain lifestyle choices. Interestingly, while noncalcified plaque build-up is driven mostly by environmental factors, the calcified plaque is more related to the genetic background of a patient [11]. Despite the significance of genetic factors, prominent risk prediction models, such as SCORE and PCE, do not explicitly account for them. Although lipoprotein(a) [Lp(a)]—a biomarker primarily determined by genetics—has been recognized as an important risk factor for ASCVD, it should be considered together with other genetic risk factors, as recent studies indicated that its contribution to CAD is orthogonal to polygenic risk score (PRS) [12,13]. To investigate whether the genetic component of risk factors alone could predict CAD or if additional signal is captured by incorporating a CAD PRS, two metaPRS models were developed and tested in this study.

Although some aspects of genetic susceptibility to CAD are captured through clinically measurable risk factors, a substantial portion remains hidden deep in the genome, beyond current clinical detection. In a study assessing pleiotropic effects of 62 CAD loci, it was found that only 24 (38.7%) showed statistical association with a traditional cardiovascular risk factor [8]. In fact, up to 30% of patients who experience a heart attack have no previously identified clinical risk factors such as HT, DM, hypercholesterolemia, or smoking history [14,15,16]. Clinical risk calculators might dismiss up to 40% of patients with high cardiovascular risk [17], which points towards the existence of factors beyond those currently taken into consideration in assessing ASCVD. In particular, this supports the liability threshold model for CAD, in which genetic and environmental factors act together to cause a disease once the threshold is reached [18].

It is vitally important for public health initiatives to identify individuals with high genetic risk and limited or absent “classical” risk factors, in order to enable preventive measures early in life. One approach to capture those individuals are PRSs. Currently, the PGS Catalog provides 71 PGSs associated with CAD or synonymous (incident coronary artery disease, coronary atherosclerosis, coronary artery disease, and coronary vascular disease) [19]. The early PGSs assessed as few as 27 variants [20], whereas the latest versions assess as many as 7M variants [21]. Based on the analysis of hundreds to millions of single-nucleotide polymorphisms (SNPs), a PRS captures the genetic risk stemming from polymorphisms scattered across the entire genome, both in coding and non-coding regions.

Significant progress is being made in understanding and integrating CAD PRSs into clinical practice [22,23,24,25]. The path toward establishing specific guidelines has begun, with advancements in the field and emerging studies steadily paving the way for widespread clinical adoption. The American Heart Association (AHA) issued a scientific statement providing guidance on the use of PRSs across a range of cardiovascular conditions [3]. In 2021, the European Society of Cardiology (ESC) acknowledged the potential of PRS in risk stratification, highlighting the need to standardize PRS for clinical use [18]. This paper aims to summarize the current state of knowledge and present new original data on cardiovascular and lipidic PRS recently developed by Allelica and clinically validated in the All of Us (AoU) dataset, a large multi-ethnic U.S. based cohort [26]. These PRSs include blood lipids such as LDL-C, HDL-C, and triglycerides, as well as conditions that serve as independent risk factors, such as HT and type 2 DM (T2DM), or share common etiology with CAD, such as atrial fibrillation (AF). While the risk of ASCVD arises from a complex interplay of multiple factors, pinpointing the dominant contributor in an individual patient is crucial for implementing targeted preventive strategies.

## 2. Materials and Methods

### 2.1. Study Design

This study utilized short-read whole-genome sequencing data from version 7 of the AoU Researcher Program, comprising a total of 245,203 individuals [26]. Individuals identified as genetically related (n = 15,359) and those with unknown sex at birth (n = 4708) were excluded, resulting in a final analysis cohort of 225,136 individuals.

AoU Research Program is a nationwide initiative that enrolls individuals aged 18 and above from across the United States. This diverse cohort contributes a wide array of data, including survey responses, physical measurements, biospecimens, and electronic health records (EHR), which encompass billing codes, prescriptions, and lab results. The program places a particular emphasis on recruiting populations that have been historically underrepresented in biomedical research. For this study, we used Controlled Tier data (C2022Q4R13) from the All of Us Researcher Workbench, focusing on participants with available ICD codes, medications, or lab results in their EHRs. In the testing dataset, age of enrollment ranged between 18 and 114 years. The majority of participants were of European genetic ancestry (48.74%), followed by African (20.27%), Admixed American (14.64), Other (not belonging to one of the other ancestries or is an admixture or Middle East, 13.11), East Asian (2.7%), and South Asian (0.97%). Women corresponded to 59.9% of the testing data.

### 2.2. Definition of Clinical Outcomes and Risk Factors

Genetic ancestry for study participants was assigned based on definitions provided by the AoU Research Program, which uses genetically inferred ancestry to categorize participants. Participants were grouped into six categories: African (AFR), Hispanic/Latino (defined as Admixed American in the current study, AMR), East Asian (EAS), European (EUR), South Asian (SAS), and Admixed (ADM). Due to the relatively small sample size of participants of Middle Eastern descent (n = 497), they were combined with the “Other” category to form the Admixed (ADM) category. Detailed descriptions of the procedures for assessing relatedness and assigning genetic ancestry can be found in the program’s documentation [26].

We used linked Electronic Health Records data to ascertain serum levels of LDL-C, HDL-C, and triglycerides, as well as clinical outcomes such as AF, T2DM, HT, and CAD. Prevalent or incident cases for clinical outcomes were defined as individuals diagnosed before or after enrollment, respectively. Only prevalent cases were used in the analysis because the follow-up period was limited to around two years, and, therefore, there was insufficient time for a meaningful number of incident cases to occur.

CAD, AF, HT, and T2DM outcomes were defined on the basis of Concept IDs or clinical diagnoses codes reported in Supplementary Tables S5–S8, respectively. Diagnosis was defined by the presence of at least 2 instances of the related ICD9 or ICD10 diagnoses codes.

Measurements of blood LDL-C in mg/dL (Table S1), HDL-C (Table S2) and triglycerides (Table S3) were selected before individual enrollment date and/or before the initiation of any lipid-lowering or DM medication (Table S4) therapy or any CAD-related outcome (Table S5). Repeated measurements for each individual were averaged. For LDL-C and triglycerides, measured values <20 or >500 mg/dL were excluded as outliers given the heightened likelihood of these extreme levels being attributed to monogenic variants, e.g., homozygous FH. For HDL-C, measured values <10 and >100 were discarded as outliers.

Binary lipid-dependent risk factors were defined on the basis of the most recent blood cholesterol guidelines [27]. LDL-dependent elevated risk: individuals with at least two measures (persistent according to guidelines) of LDL-C levels ≥160 mg/dL were considered having this risk factor (N. 60,938).

LDL-dependent high risk: individuals with at least two measures of LDL-C levels ≥190 mg/dL were considered to have this risk factor (N. 60,938).

Triglycerides-dependent elevated risk: individuals with at least two measures of triglycerides levels ≥175 mg/dL were considered having this risk factor (N. 62,857).

HDL-dependent elevated risk: individuals with at least two measures of HDL-C < 40 mg/dL (N. 61,672)

### 2.3. PRS Development

Lipid-specific as well as PRS for AF, T2DM, and HT were developed using Allelica’s DISCOVER V1.3 software, which applies a battery of different PRS algorithms to input summary statistics in order to yield a range of trait-specific PRSs. The best performing trait-specific PRS is then identified using a validation dataset. The performance of the best performing PRS are further confirmed in a final testing dataset. LDL-C, HDL-C, and triglyceride PRS were generated from GWAS data [28], similarly as PRSs for AF [29], T2DM [30], and HT [31].

Each best performing trait-specific PRS was identified among multiple algorithm-generated PRSs in the validation dataset by means of logistic regression. Each ancestry-adjusted PRS corresponded to the prediction variable, while age, sex and first 4 principal components of ancestry were used as control covariates with binary disease outcomes as the response variable. The best performing trait-specific PRS was defined on the basis of the PRS odds ratio per standard deviation (OR per SD). The first release of UK Biobank (N. 136,653) was used as the validation dataset. Prediction performance of each best performing trait-specific PRS were subsequently confirmed in ancestry-specific subgroups of the second UK Biobank release (testing dataset, N. 349,669)

Two meta PRS were developed: (i) *risk factors metaPRS* integrates all the PRSs included in the Allelica Cardiovascular Panel comprising HT (Allelica_BP_vI), T2DM (Allelica_T2D_vI), AF (Allelica_AF_vI), HDL-C (Allelica_HDL_vI), LDL-C (Allelica_LDL_vI), triglycerides (Allelica_TG_vI), and Lp(a) (PGS000667) and (ii) *risk factors* + *CAD metaPRS* by integrating the ancestry-specific CAD PRSs from Busby et al. [24] and a multi-ancestry CAD PRS (PGS003725) [32] into the *risk factors metaPRS*. Ancestry- and PRS panel-specific contributions (panel betas) were estimated and tested separately within each ancestry group in the AoU cohort. For each ancestry, the cohort was divided into 5 subsets, with 4/5 of the individuals used for estimating the panel-specific betas and 1/5 reserved for testing, following a 5-fold cross-validation approach. Logistic regression was employed for this analysis, controlling for the first four principal components, age, and sex. The PRS panel constituents were subsequently integrated by summing the effect sizes of each panel, weighted by their respective PRS panel-specific betas.

### 2.4. Ancestry Specific Clinical Distributions and Ancestry Adjustment of PRSs

After calculating individual-level PRSs, adjustments for ancestry and normalization were applied. To account for ancestry, a well-documented method [33] was used, which involves removing the influence of the first four principal components from the PRS values (as outlined in Equations (1) and (2) below). This adjustment was conducted separately for each ancestry-specific subset of the AoU cohort. Normalization ensured that the PRSs were standardized, yielding a mean of zero and a standard deviation of one.

(1) $$Adjusted\;Model_{ANC}\;:\;PRS_{raw}\;\sim \;PC_{1}+PC_{2}+PC_{3}+PC_{4}$$

(2) $$PRS_{adj}=PRS_{raw}-Adjusted\;Model_{ANC}\;\left(PC_{1}+PC_{2}+PC_{3}+PC_{4}\right)$$

### 2.5. Data Analysis

All analyses were carried out in the Researcher Workbench cloud-based platform of AoU research project. Individual level PRS were computed using plink2 (v2.00a6LM 64-bit Intel—6 August 2024). All analyses were carried out using python3 (v3.10.12). Logistic regression analyses used to estimate ancestry-specific and PRS-specific weights for META PRS generation and to estimate odds ratio per standard deviation were performed using the logit function from the statsmodels (v0.14.4) Python package. Cross-validation and Area Under the Curve were performed using the KFold and roc_auc_score functions, respectively, from the sklearn Python package. All plots were generated using the Python packages matplotlib.

## 3. Results

### 3.1. Association of PRSs with High Lipid States

In the AoU dataset, the OR per SD for the association of the Allelica_LDL_vI PRS to the LDL-C ≥ 160 mg/dL outcome varies by genetic ancestry, ranging from 1.6 (95% CI: 1.5–1.7) in individuals of African ancestry to 2.0 (95% CI: 1.9–2.1) in those of European ancestry (Table 1). This means that the Allelica_LDL_vI PRS can identify at least 6.5% of the African ancestry subpopulation and more than 15% of the European ancestry subpopulation in AoU who have at least twice the risk (with respect to the average risk of the population) of developing an LDL-associated cardiovascular risk-enhancing factor. Even stronger risk stratification is observed when considering a threshold of LDL-C ≥ 190 mg/dL, which is an indication for statin initiation per se [34]. In this case, the Allelica_LDL_vI PRS shows OR per SD ranging from 1.9 (95% CI: 1.6–2.3) in individuals of African ancestry to 4.5 (9% CI: 1.6–12.8) in those of South Asian ancestry.

For the remaining lipidic clinical outcomes of HDL-C < 40 mg/dL and triglycerides ≥ 175 mg/dL, the corresponding PRSs are able to identify substantial subpopulation fractions with 2-fold increased risk with respect to average in all genetic ancestry subpopulations (~1–9%).

### 3.2. Association of PRSs with AF, T2DM and HT

Association of PRS (Allelica_AF_vI) with AF, as quantified by the OR per SD, ranges between 1.2 (95% CI: 1.1–1.3) for individuals of African ancestry to 1.8 (95% CI: 1.3–2.4) in East Asian individuals (Table 2).

T2DM genetic risk stratification of Allelica_T2D_vI PRS ranges between 1.2 (95% CI: 1.2–1.2) for individuals of African ancestry to 1.6 (CI 95%: 1.4–1.9) in South Asian individuals.

Risk stratification of HT of Allelica_HT_PRS ranges between 1.1 (95% CI: 1.1–1.2) for individuals of African genetic ancestry to 1.5 (95% CI: 1.3–1.8) in South Asian individuals.

### 3.3. Association of Two metaPRS with CAD

To assess the effect of incorporating the genetics of CAD risk factors in a metaPRS to predict CAD, we generated a new metaPRS, which combines PRS for lipids (LDL-C, HDL-C, triglycerides, Lp(a)) and cardiometabolic diseases (HT, T2DM, and AF) called *risk factors metaPRS.* We also generated another metaPRS, called *risk factors* + *CAD metaPRS*, that integrates two multi-ancestry CAD PRS: PGS003725 and Allelica CAD PRS [24] with the *risk factor metaPRS.*

In the AoU cohort, the *risk factor metaPRS* displayed significant risk stratification in each of the populations analyzed (Table 3). The *risk factor* + *CAD metaPRS* showed higher predictive performance across all the ancestries analyzed with an improvement in OR per SD ranging from 46% in SAS to about 10% in AMR. The percentage of the total population having at least twice the average risk of CAD serves as a good measure of *the risk factors* + *CAD metaPRS* effectiveness. Stronger risk stratification using the *risk factors* + *CAD metaPRS* is observed in the SAS population, where more than 25% of the individuals were identified as having at least twice the average risk. Significant proportions of high-risk participants were also identified in EUR (9.6%), EAS (7%), AMR (4.4%), and ADM (4.4%) ancestries. In contrast, a lower percentage of the high-risk population was detected in the African (AFR) ancestry (0.4%)

### 3.4. Impact of Ancestry on PRS Models

**Figure 2:**
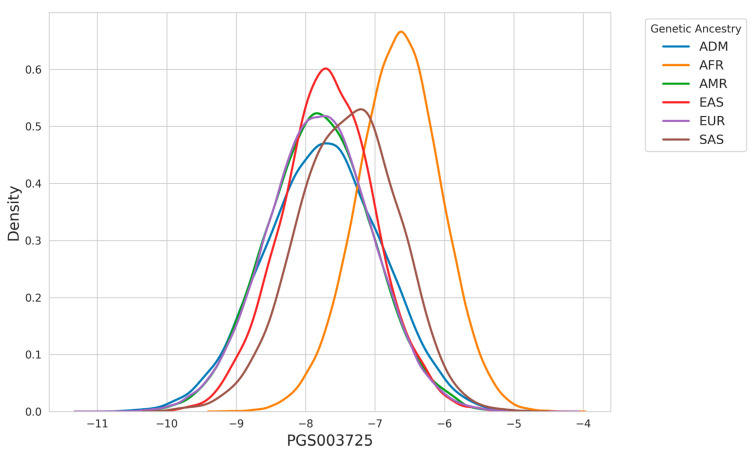
Density distribution of PGS003725 across genetic ancestries. The plot highlights differences in the distribution of PGS003725 scores among ancestries, with individuals of AFR ancestry displaying a higher peak at higher score values compared to other groups.

The effect of differences in allele frequencies and linkage disequilibrium patterns among various ancestry groups on risk prediction accuracy in PRS models is well-documented [35]. This variability posed a significant challenge to the clinical adoption of PRS models in their earlier stages of development. However, the incorporation of ancestry adjustment techniques (as detailed in the Section 2) has substantially mitigated these issues [24]. To demonstrate the significance of this adjustment in PRS calculation, Figure 2 illustrates the ancestry-specific distribution of the PGS003725 score. The score is derived by multiplying the effect size of each variant by the allele count of each individual, providing a raw calculation of genetic risk.

**Figure 3:**
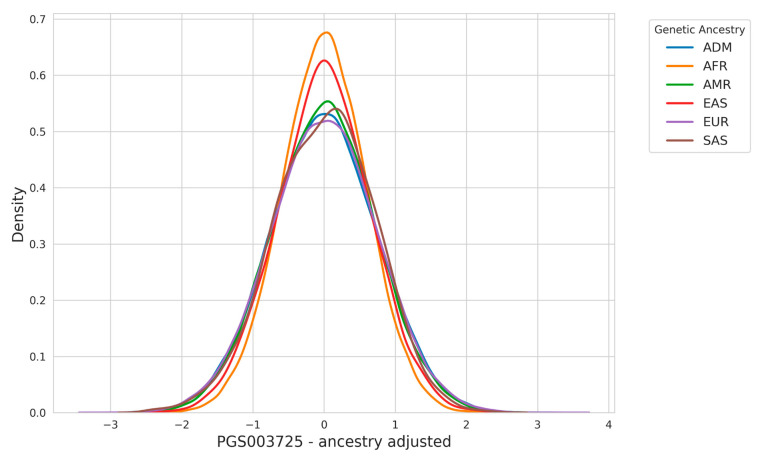
Ancestry adjustment ensures comparable PRS distributions across populations.

In Figure 3, we present the outcomes of applying the ancestry adjustment methodology described in the Section 2, which disentangles the confounding effects of principal component analysis (PCA)-derived population structure from the final PRS calculation. This approach addresses the biases introduced by population stratification, where differences in genetic architecture across ancestry groups can affect risk predictions.

The adjustment process involves calculating a residual PRS by regressing out the effects of PCA-based ancestry components from the raw PRS values. Specifically, the raw PRS, which is derived by summing the product of variant effect sizes and individual allele counts, is corrected using a linear model that incorporates ancestry covariates such as principal components. By removing these confounding factors, the adjusted PRS isolates the polygenic contribution to disease risk that is independent of ancestry-driven allele frequency differences.

The results in Figure 3 highlight how the ancestry adjustment substantially narrows the difference observed across ancestry groups in the raw PRS distribution.

The ancestry adjustment process ensures that PRS distributions are comparable across populations while accounting for fine-scale population structure, minimizing biases due to differences in genetic backgrounds. However, accurate risk estimation requires more than aligned PRS distributions as ancestry specific effect sizes need to be estimated. To address this, we developed ancestry-specific risk models that incorporate both genetic and population-specific factors.

### 3.5. Model Calibration

Calibration evaluates the agreement between predicted probabilities and observed event rates, providing a measure of model reliability. For this study, we employed a binning-based calibration methodology. Predicted probabilities for each trait or outcome were divided into equally spaced bins (quintiles of predicted risk distributions), and the observed event rates within each bin were calculated. The 95% confidence intervals (CI) for these observed rates were estimated using binomial proportion methods. A spline interpolation was applied to the observed rates across bins, creating a calibration curve. The diagonal line in the calibration plots represents perfect calibration, where predicted probabilities match observed outcomes exactly. Deviations from this line indicate systematic biases: upward deviations reflect under-prediction (observed rates exceed predictions), while downward deviations indicate over-prediction.

Multi-ancestry evaluation examines model performance across diverse populations, ensuring the predictions are valid and unbiased for each ancestry. Instead of assuming uniform applicability, this approach tests the model’s ability to account for population-specific genetic and environmental variability. Ancestral groups often differ in key parameters such as allele frequencies, linkage disequilibrium patterns, genetic architecture, and trait prevalence. These differences can result in prediction biases when a model trained predominantly on one ancestry (e.g., European) is applied to others (e.g., African or South Asian). A multi-ancestry evaluation enables the detection of such biases by assessing calibration performance separately for each group, highlighting specific instances where the model fails to generalize or systematically underperforms.

Calibration plots illustrate the performance of predictive models across six ancestral groups: African (AFR), Admixed American (AMR), East Asian (EAS), European (EUR), Admixex (ADM), and South Asian (SAS). The x-axis represents the mean predicted probability for each bin, while the y-axis shows the observed event rate within the same bin.

The diagonal line in each panel denotes perfect calibration, while the blue points represent observed event rates with 95% CI shown as error bars. A blue spline-smoothed calibration curve connects the observed rates, providing a continuous representation of calibration trends. Discrepancies between the spline and the diagonal line indicate deviations from ideal predictions, where over- or under-estimation of risk may occur.

**Figure 4:**
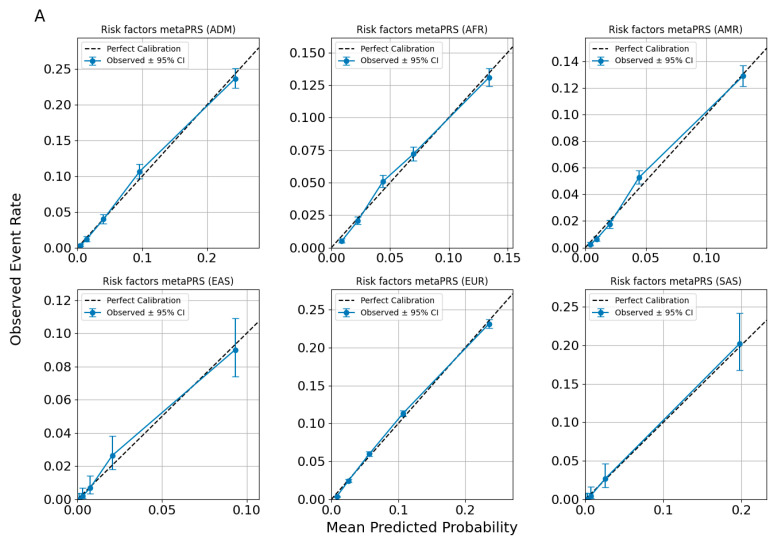

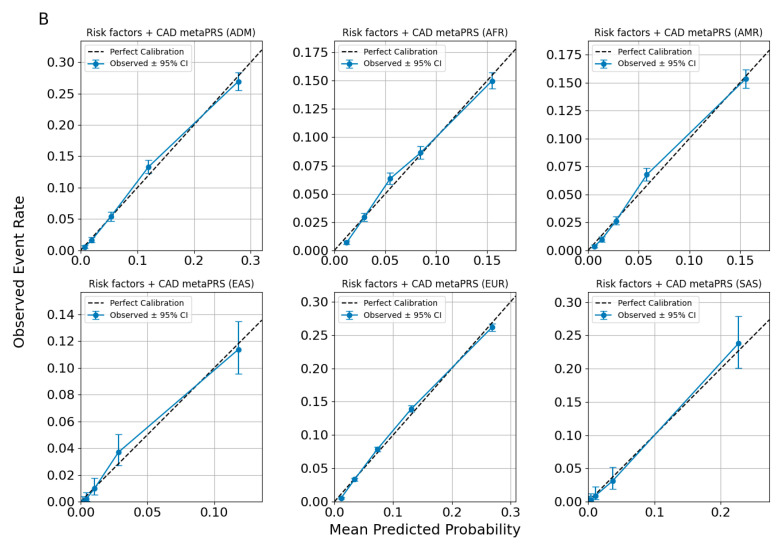
Calibration plots for CAD predictions using ancestry-adjusted *risk factors metaPRS* (**A**) and *risk factors* + *CAD metaPRS* models (**B**).

Figure 4 evaluates different sets of traits to assess the calibration performance of predictive models across ancestries. Figure 4 specifically focuses on the calibration of the Allelica CAD metaPRS. Cardiometabolic traits (AF, HT, T2DM) and lipid-related traits (LDL > 160 mg/dL, HDL, and triglycerides) are presented in the Supplementary Figure (Figure S1).

Across all figures, the calibration patterns appear consistent for each disease or trait within each ancestry, with no evidence of systematic miscalibration. The alignment of the spline curves with the diagonal line indicates that the models maintain similar predictive performance across ancestries for the evaluated traits. Observed deviations tend to be limited to specific probability ranges and are not uniformly present across all ancestries or traits, suggesting that the models are broadly well-calibrated without significant ancestry-specific biases.

**Figure 5:**
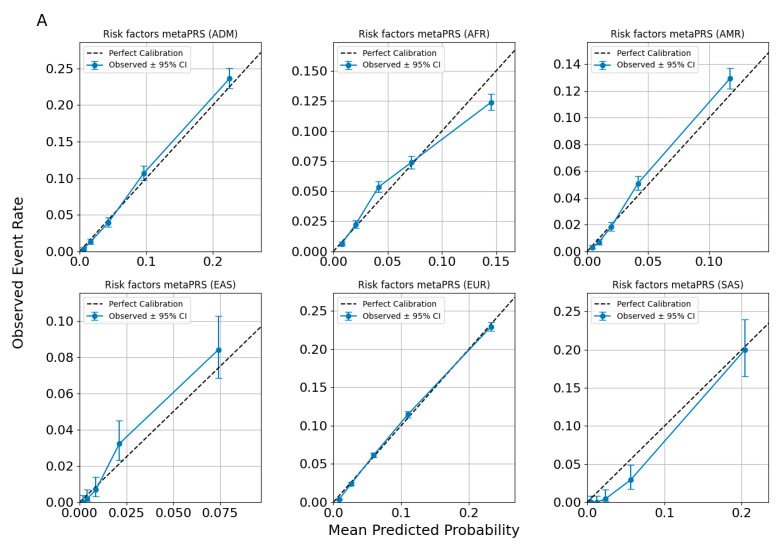

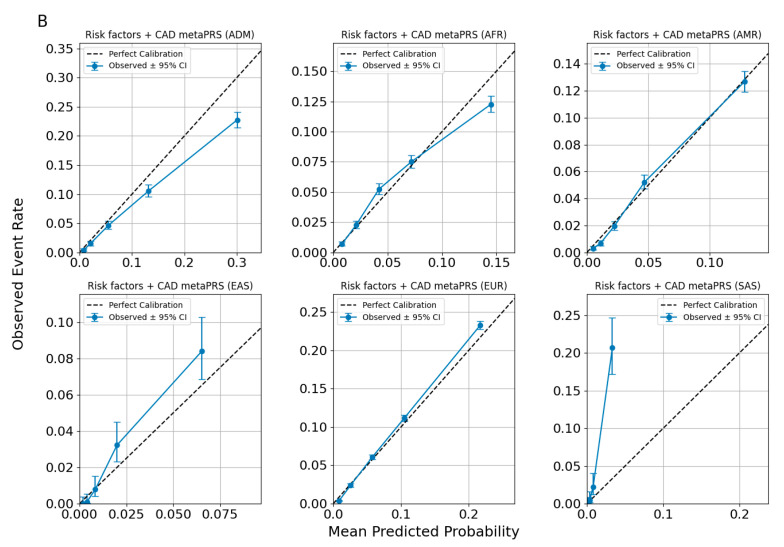
Calibration plots for CAD predictions using *risk factors metaPRS* (**A**) and *risk factors* + *CAD* Allelica CAD metaPRS (**B**) models. developed without accounting for ancestry.

In Figure 5, we present a calibration plot for the Allelica CAD metaPRS models under the assumption of non-ancestry-adjusted and non-ancestry-specific risk-building approaches. The results demonstrate that the model underestimates risk for South Asian (SAS) individuals and overestimates risk for individuals of African (AFR) ancestry. Notably, the only population where the risk remains calibrated is the European (EUR) population. These findings highlight the critical importance of accounting for the specificity of each ancestry group when implementing genetic background-based tests in precision medicine.

## 4. Discussion

In this paper, we presented multiple PRSs designed for the individual assessment of the genetic predisposition of lipid levels and cardiometabolic diseases. Each of these PRS has the potential to contribute to precision medicine, particularly in guiding preventive strategies and personalized healthcare interventions. The two big advantages of PRSs over most other established risk factors and risk modifiers is that they can be performed once-in-a-lifetime and pre-emptively.

The primary goal of using PRSs for CAD is to identify at-risk patients who are not detected by traditional risk models. The 10-year ASCVD risk, estimated via the standard PCE, has a strikingly similar 10-year risk distribution in groups of patients with high vs. low CAD PRS [24,36]. This highlights that PRS captures an element of ASCVD risk, which is orthogonal to currently established clinically measurable risk factors. Notably, individuals with a high PRS and non-elevated LDL-C levels have a comparable risk of ASCVD to that in individuals with LDL-C > 190 mg/dL and an average PRS, even when the model is controlled for other risk factors [37]. This underscores the utility of PRSs in identifying high-risk individuals who might otherwise remain undetected through traditional metrics. The significance of PRS is particularly pronounced in relatively young individuals, whose limited exposure to environmental risk factors due to their age means that a greater proportion of their risk stems from intrinsic, genetic factors. Furthermore, these individuals often have not yet developed clinical risk indicators, making traditional risk assessments less effective. In a study using a CAD PRS comprising 241 SNVs, myocardial infarction was more strongly associated with high CAD PRS in younger age groups. Specifically, in patients younger than 50 years, high PRS resulted in 1.7 HR per SD, compared to 1.5 in the 50–60 year-old and older than 60 year age groups [38]. Integrating traditional risk assessments with PRSs has the potential to ensure a more comprehensive identification of high-risk populations, thereby improving prevention and management strategies.

The clinical utility of CAD PRS lies in its ability to enable clinical decisions that lead to improved health outcomes. A study found that incorporating a high CAD PRS into recommendations for statin therapy could result in the treatment of an extra 4.1% of the entire primary prevention population [36]. Also, when the 10-year rate of atherosclerotic cardiovascular disease was assessed in groups of patients divided into subgroups according to their CAD-PRS and clinical risk combined, it was found that 20% of patients with borderline clinical risk and a high PRS had a higher 10-year ASCVD risk, putting them into the risk category requiring statin therapy. On the other hand, 20% of patients from borderline and intermediate clinical risk and low genetic risk had a 10-year ASCVD rate low enough to delay the initiation of statin therapy. However, the authors pointed out that in patients with low clinical 10 year risk of ASCVD (<5%) or patients older than 70 years old, a CAD PRS will most likely not change a therapeutic strategy. For these groups, PRS assessment may be less beneficial [38].

A randomized controlled trial, MI-GENES, proved that adding PRS to Framingham risk score assessments in patients with intermediate risk of CHD resulted in higher statin initiation rate, lower LDL-C, and lower rate of major adverse cardiovascular events over a median period of 9.5 years [39]. Interestingly, there is also data showing that patients with high CAD-PRS might benefit more from hypolipidemic therapies than the rest of the population [37,40,41], and a study showed that 20% of patients with the highest PRS score had a 46% risk reduction when on statins compared to 26% in the remaining 80% [40]. In a study aimed to assess benefits from PCSK9 inhibitor evolocumab, high genetic CAD risk resulted in the biggest clinical benefit from the treatment regardless of the clinical risk [41]. Genetic data obtained for CAD PRS assessment could also guide personalized hypolipidemic strategies independently of the PRS, potentially enhancing treatment efficacy and reducing adverse effects, as a growing body of evidence explains individual variation in response to therapy [42,43,44,45,46,47,48,49]. Identifying the dominant contributors to an individual’s cardiovascular risk enables proactive health management. For example, a patient with a genetic tendency toward elevated LDL-C can focus on strategies to lower its levels, while someone at higher genetic risk for T2DM can prioritize maintaining optimal blood glucose control. On an individual level, this personalized approach empowers individuals to adopt tailored lifestyle and dietary interventions that address their specific risks. On a populational level, stratifying patients can enhance their characterization and allow for the identification of previously unnoticed nuances, such as varied responses to treatments, or susceptibility to complications advancing precision medicine.

A structured workflow incorporating CAD PRS into clinical practice could begin with applying a traditional ASCVD risk model followed by ordering a CAD PRS for individuals who may benefit, as described above. Next, a standard blood sample or a buccal swab from a patient is collected and forwarded to a genetic laboratory for a single nucleotide polymorphism (SNP) array or whole genome sequencing (WGS) followed by the computational CAD PRS analysis. The PRS report, which provides a CAD risk assessment, is then delivered to the ordering clinician. The results may reveal previously unrecognized CAD risk (e.g., in young individuals without traditional risk factors) or refine risk assessment by reclassifying patients into higher risk categories, ultimately influencing treatment decisions. Additionally, a PRS for key genetic contributors to cardiovascular risk (e.g., LDL-C, T2DM) can be derived from the same genetic data, further guiding preventative or therapeutic strategies. The cost-effectiveness of this approach can vary significantly depending on the healthcare system, specifically factors such as accessibility and cost of physician visits or routine check-ups (e.g., blood lipid levels). The existing analyses suggest that incorporating PRSs into CAD risk assessment provides economic benefits [25,50,51], making this approach valuable even in resource-limited settings, where a one-time investment in a PRS assessment could help reduce future healthcare costs. Accelerating the insurance reimbursement pathway is also crucial, as it would significantly enhance equitable access to PRS testing and allow broader populations to benefit from the preventative and personalized healthcare insights these tests provide.

One of the key challenges in integrating PRS into clinical practice at scale is educating clinicians about its utility while addressing concerns about workflow burden in an already demanding healthcare environment. To encourage adoption, it must be clear that PRS is a simple, high-value tool that seamlessly integrates into existing risk assessment frameworks. Additionally, clinicians need to be well-equipped to communicate the benefits and limitations of PRS to patients, just as they would with any other genetic test. Providing clear, patient-friendly educational materials is essential to ensure informed decision-making. Importantly, PRS tests have now evolved into clinical-grade diagnostic tools that can be ordered as easily as routine genetic tests, reducing barriers to adoption and minimizing additional workload for physicians.

A key criticism of PRSs was their potential to exacerbate healthcare inequalities. This arose because genomic data have historically been disproportionately derived from individuals of European ancestry, and transferring PRS developed on this basis across populations was challenging [35,52]. However, there have been examples of successful solutions to this problem [32], and a multi-ancestry PRS we developed previously showed clinical utility as a risk enhancer across five genetic ancestry groups (African, Admixed American, East Asian, European, South Asian) and admixed individuals [24]. These ancestry-specific CAD PRSs optimized for different genetic ancestry groups were able to identify a significant portion of a population with twice the risk of the remainder. The percentage of population falling into that category varied depending on the ancestry (12–24%). It is important to acknowledge that although PRS performance varies across ancestry groups, validated and calibrated PRSs are available across all major continental level ancestry groups, representing an important advancement compared to traditional risk models, such as the PCE, that under [5] or overestimate risk [53] in non-European population. In this study, we validated our multi-ancestry PRSs using data from the recently released U.S. prospective study, the AoU cohort. The presented results demonstrate that incorporating ancestry-specific characteristics is no longer a challenge to overcome, but rather a valuable resource, enabling precision medicine to be applied equitably across diverse populations.

Another limitation of the current study is the cross-sectional nature of the analysis, combined with a relatively short median follow-up period of approximately two years, which constrains our ability to assess the long-term predictive value of PRS for clinical outcomes. The small number of incident cases, resulting from the limited follow-up duration, further restricts our capacity to evaluate the predictive power of PRS in terms of incident survival dynamics. Moreover, challenges in identifying incident cases may have led to an underestimation of the PRSs’ risk stratification performance (OR per SD) as individuals labeled as controls will develop CAD as the follow up increases and therefore they will be labeled as cases. This is further supported by evidence indicating lower predictive performance in AoU compared to other prospective studies [54]. However, as the AoU cohort continues to grow, future studies will benefit from extended follow-up, a larger sample size, and greater ancestral diversity. These advancements may help overcome current limitations and enable a more comprehensive reassessment of PRS predictive performance in the years ahead.

A further constraint was highlighted in a recent study [55], which showed that the classification of individuals in the top 10% of 57 CAD PRS varied widely, suggesting substantial instability in using PRSs to classify individuals into high-risk categories. However, the same study demonstrated that integrating multiple PRSs panels into a single score significantly attenuated this instability. In the current study, we aimed to reduce potential instability by integrating two multi-ancestry CAD PRS, which demonstrated strong risk stratification performance, along with PRSs for CAD risk factors and cardiometabolic outcomes, using a META PRS approach. This study demonstrated improved prediction when CAD PRS was incorporated into the *risk factor metaPRS*, highlighting the presence of a genetic component in CAD etiopathology that extends beyond traditional risk factors, whether directly measured or genetically determined. To evaluate this, two metaPRS models were developed and tested: the *risk factor metaPRS*, which combines PRS for lipids (LDL-C, HDL-C, triglycerides, Lp(a)), as well as cardiometabolic diseases (HT, T2DM, AF), and the *risk factor* + *CAD metaPRS*, which integrates two multi-ancestry CAD PRSs with the *risk factor metaPRS*. In the AoU cohort, the *risk factor metaPRS* demonstrated significant risk stratification across all analyzed populations. However, its effect size may be underestimated due to the limited follow-up period, during which events may not have fully developed or had enough time to occur, resulting in some cases still being labeled as controls.

## 5. Conclusions

Here, we report the validation of a *risk factor* + *CAD metaPRS* in a cohort from the AoU initiative, which became available approximately five years ago. We demonstrate the efficacy of combining genetic signals from several related clinical conditions to improve CAD risk prediction. The *risk factor* + *CAD metaPRS* was developed by integrating previously published multi-ancestry CAD PRS with additional PRS for CAD risk factors and other cardiometabolic diseases. This PRS exhibited good risk stratification performance. We also discuss the nuances of risk prediction across a diverse genetic ancestry spectrum and emphasize the importance of ancestry adjustment for proper risk calibration.

The Allelica suite of cardiometabolic and lipid PRSs represents an important step forward in precision medicine for the prevention and management of common disease. By integrating comprehensive genetic insights from CAD-specific PRS with PRSs for lipid traits (LDL-C, HDL-C, triglycerides) and related cardiometabolic conditions (T2DM, HT, AF), this tool provides a robust, multi-ancestry framework for personalized risk stratification.

The clinical implications are significant: PRS-guided strategies facilitate earlier and more precise identification of high-risk individuals, allowing for tailored interventions targeting specific pathways, such as lipid management and lifestyle modifications. This multi-trait approach ensures a comprehensive risk assessment that aligns genetic predisposition with actionable clinical factors, ultimately enhancing the precision of prevention strategies for cardiovascular disease in diverse populations.

## Figures and Tables

**Table 1:** Association (Odds Ratio per Standard Deviation: OR per SD) and discrimination (Area Under the Curve: AUC) performance of ancestry-specific polygenic risk scores (PRSs) with high-risk lipid status (outcome) in different ancestry groups (African: AFR, Admixed American: AMR, East Asian: EAS, European: EUR, South Asian: SAS, Admixed: ADM). The PRS column refers to the polygenic risk score, the AUC (95% CI) column shows the Area Under the Curve with 95% Confidence Intervals, and the OR per SD (95% CI) column reports the Odds Ratio per standard deviation of PRS with 95% Confidence Intervals. The Outcome column represents the case/control classification definition, and the Ancestry column indicates the genetic ancestry group. Cases and Controls refer to the number of cases and controls in each ancestry group. Lipid-specific PRSs are as follows: Allelica_LDL_vI for high LDL, Allelica_HDL_vI for low HDL, and Allelica_TG_vI for high triglycerides.

PRS	Outcome	Ancestry	Cases	Controls	AUC (95% CI)	OR per SD (95% CI)
Allelica_LDL_vI	LDL-C ≥ 160	AFR	642	10,476	0.68 (0.66–0.7)	1.6 (1.5–1.7)
Allelica_LDL_vI	LDL-C ≥ 160	AMR	331	7218	0.73 (0.7–0.75)	1.8 (1.6–2.1)
Allelica_LDL_vI	LDL-C ≥ 160	EAS	80	1093	0.72 (0.67–0.77)	1.9 (1.5–2.5)
Allelica_LDL_vI	LDL-C ≥ 160	EUR	2587	32,716	0.71 (0.7–0.72)	2.0 (1.9–2.1)
Allelica_LDL_vI	LDL-C ≥ 160	ADM	382	4825	0.69 (0.66–0.71)	1.6 (1.5–1.8)
Allelica_LDL_vI	LDL-C ≥ 160	SAS	37	551	0.73 (0.64–0.81)	1.9 (1.3–2.8)
Allelica_LDL_vI	LDL-C ≥ 190	AFR	145	10,973	0.74 (0.7–0.77)	1.9 (1.6–2.3)
Allelica_LDL_vI	LDL-C ≥ 190	AMR	53	7496	0.72 (0.64–0.8)	1.9 (1.4–2.6)
Allelica_LDL_vI	LDL-C ≥ 190	EAS	13	1160	0.85 (0.74–0.93)	2.7 (1.4–5.3)
Allelica_LDL_vI	LDL-C ≥ 190	EUR	394	34,909	0.75 (0.73–0.77)	2.4 (2.1–2.7)
Allelica_LDL_vI	LDL-C ≥ 190	ADM	69	5138	0.73 (0.67–0.78)	2.0 (1.6–2.6)
Allelica_LDL_vI	LDL-C ≥ 190	SAS	7	581	0.87 (0.79–0.95)	4.5 (1.6–12.8)
Allelica_HDL_vI	HDL-C < 40	AFR	1371	9531	0.62 (0.61–0.64)	1.2 (1.2–1.3)
Allelica_HDL_vI	HDL-C < 40	AMR	1287	6553	0.65 (0.63–0.67)	1.4 (1.3–1.5)
Allelica_HDL_vI	HDL-C < 40	EAS	101	1083	0.76 (0.70–0.8)	1.7 (1.3–2.1)
Allelica_HDL_vI	HDL-C < 40	EUR	4650	31,303	0.72 (0.71–0.73)	1.6 (1.5–1.6)
Allelica_HDL_vI	HDL-C < 40	ADM	680	4518	0.72 (0.70–0.74)	1.4 (1.3–1.6)
Allelica_HDL_vI	HDL-C < 40	SAS	98	497	0.75 (0.70–0.8)	1.3 (1.0–1.7)
Allelica_TG_vI	Trigs-C ≥ 175	AFR	910	9980	0.62 (0.60–0.64)	1.2 (1.1–1.2)
Allelica_TG_vI	Trigs-C ≥ 175	AMR	1230	6638	0.63 (0.61–0.65)	1.4 (1.3–1.5)
Allelica_TG_vI	Trigs-C ≥ 175	EAS	163	1014	0.71 (0.67–0.75)	1.4 (1.1–1.6)
Allelica_TG_vI	Trigs-C ≥ 175	EUR	5186	31,873	0.65 (0.64–0.66)	1.5 (1.5–1.6)
Allelica_TG_vI	Trigs-C ≥ 175	ADM	672	4598	0.67 (0.65–0.69)	1.5 (1.4–1.6)
Allelica_TG_vI	Trigs-C ≥ 175	SAS	91	502	0.73 (0.68–0.78)	1.6 (1.2–2.0)

**Table 2:** Association (Odds Ratio per Standard Deviation: OR per SD) and discrimination (Area Under the Curve: AUC) performance of polygenic risk scores (PRSs) with clinical outcomes (outcome) in different ancestry groups (African: AFR, Admixed American: AMR, East Asian: EAS, European: EUR, South Asian: SAS, Admixed: ADM). The PRS column refers to the polygenic risk score, the AUC (95% CI) column shows the Area Under the Curve with 95% Confidence Intervals, and the OR per SD (95% CI) column reports the Odds Ratio per standard deviation of PRS with 95% Confidence Intervals. The Outcome column represents the case/control classification. The Ancestry column indicates the genetic ancestry group. Cases and Controls refer to the number of cases and controls in each ancestry group. Clinical outcomes are as follows: Atrial Fibrillation (AF), Type 2 Diabetes Mellitus (T2DM), and Hypertension (HT). PRSs reported in the table are as follows: Allelica_AF_vI for Atrial Fibrillation, Allelica_T2D_vI for Type 2 Diabetes mellitus, and Allelica_BP_vI for HyperTension.

PRS	Outcome	Ancestry	Cases	Controls	AUC (95% CI)	OR per SD (95% CI)
Allelica_AF_vI	AF	AFR	701	46,070	0.75 (0.73–0.77)	1.2 (1.1–1.3)
Allelica_AF_vI	AF	AMR	462	35,755	0.81 (0.79–0.83)	1.3 (1.2–1.4)
Allelica_AF_vI	AF	EAS	53	5051	0.88 (0.85–0.91)	1.8 (1.3–2.4)
Allelica_AF_vI	AF	EUR	5451	107,002	0.82 (0.82–0.83)	1.6 (1.5–1.6)
Allelica_AF_vI	AF	ADM	603	17,549	0.84 (0.83–0.85)	1.5 (1.4–1.6)
Allelica_AF_vI	AF	SAS	19	2218	0.86 (0.76–0.93)	1.6 (1.0–2.5)
Allelica_T2D_vI	T2DM	AFR	6221	40,138	0.7 (0.69–0.7)	1.2 (1.2–1.2)
Allelica_T2D_vI	T2DM	AMR	4621	31,190	0.75 (0.75–0.76)	1.4 (1.4–1.45)
Allelica_T2D_vI	T2DM	EAS	278	4802	0.78 (0.76–0.8)	1.4 (1.2–1.6)
Allelica_T2D_vI	T2DM	EUR	11,137	102,965	0.69 (0.69–0.7)	1.5 (1.5–1.6)
Allelica_T2D_vI	T2DM	ADM	1788	16,453	0.74 (0.73–0.75)	1.4 (1.4–1.5)
Allelica_T2D_vI	T2DM	SAS	192	2023	0.83 (0.81–0.86)	1.6 (1.4–1.9)
Allelica_BP_vI	HT	AFR	12,664	33,173	0.73 (0.72–0.73)	1.1 (1.1–1.2)
Allelica_BP_vI	HT	AMR	6883	28,653	0.8 (0.79–0.8)	1.4 (1.4–1.4)
Allelica_BP_vI	HT	EAS	581	4462	0.81 (0.8–0.83)	1.4 (1.2–1.5)
Allelica_BP_vI	HT	EUR	32,263	79,952	0.76 (0.75–0.76)	1.5 (1.5–1.5)
Allelica_BP_vI	HT	ADM	4480	13,509	0.8 (0.79–0.81)	1.4 (1.4–1.5)
Allelica_BP_vI	HT	SAS	291	1914	0.88 (0.86–0.89)	1.5 (1.3–1.8)

**Table 3:** Risk stratification (Odds Ratio per Standard Deviation: OR per SD) and discrimination (Area Under the Curve: AUC) performance for the association of *risk factors metaPRS* and risk factors + CAD metaPRS with CAD in different ancestry groups (African: AFR, Admixed American: AMR, East Asian: EAS, European: EUR, South Asian: SAS, Admixed: ADM). The PRS column indicates the polygenic risk score applied, the AUC (95% CI) column shows the Area Under the Curve with 95% Confidence Intervals, and the OR per SD (95% CI) column reports the Odds Ratio per Standard Deviation of PRS with 95% Confidence Intervals. The Ancestry column indicates the genetic ancestry group. Cases and Controls refer to the number of cases and controls in each group. Two PRSs are reported in the table: (1) risk factors metaPRS, which combines the following PRSs: Allelica_BP_vI, Allelica_T2D_vI, Allelica_AF_vI, Allelica_HDL_vI, Allelica_LDL_vI, Allelica_TG_vI, Lp(a) PRS (PGS000667), and (2) risk factors + CAD metaPRS, which combines the previously developed PRS [24] with a multi-ancestry CAD PRS (PGS003725) [32].

PRS	Ancestry	Cases	Controls	AUC (95% CI)	OR per SD (95% CI)
*risk factors metaPRS*	AFR	2609	44,027	0.74 (0.73–0.75)	1.2 (1.1–1.2)
*risk factors metaPRS*	AMR	1501	34,567	0.82 (0.81–0.83)	1.4 (1.3–1.4)
*risk factors metaPRS*	EAS	128	4976	0.87 (0.84–0.89)	1.5 (1.2–1.8)
*risk factors metaPRS*	EUR	9824	103,627	0.79 (0.79–0.79)	1.3 (1.3–1.4)
*risk factors metaPRS*	ADM	1447	16,706	0.83 (0.82–0.84)	1.3 (1.2–1.4)
*risk factors metaPRS*	SAS	104	2121	0.92 (0.91–0.94)	2.0 (1.6–2.5)
*risk factors + CAD metaPRS*	AFR	2609	44,027	0.75 (0.74–0.75)	1.3 (1.2–1.3)
*risk factors + CAD metaPRS*	AMR	1501	34,567	0.82 (0.81–0.83)	1.5 (1.5–1.6)
*risk factors + CAD metaPRS*	EAS	128	4976	0.87 (0.84–0.89)	1.6 (1.3–1.9)
*risk factors + CAD metaPRS*	EUR	9824	103,627	0.81 (0.8–0.81)	1.7 (1.7–1.8)
*risk factors + CAD metaPRS*	ADM	1447	16,706	0.83 (0.82–0.84)	1.5 (1.4–1.6)
*risk factors + CAD metaPRS*	SAS	104	2121	0.94 (0.92–0.95)	2.9 (2.2–3.8)

## Data Availability

The data utilized in this study are available from the All of Us Research Program, a project of the National Institutes of Health (NIH). Researchers can access the data through the All of Us Researcher Workbench. For more information on accessing the data, visit the All of Us Research Program website at https://allofus.nih.gov.

## Supplementary Materials

The following supporting information can be downloaded at: https://www.mdpi.com/article/10.3390/nu17050926/s1, Figure S1: Cardiometabolic traits (AF, HT, T2DM) and lipid-related traits (LDL > 160 mg/dL, HDL, and triglycerides) assessed for calibration performance across ancestry specifics predictive models; Tables S1–S8: Concept IDs and corresponding descriptions used to identify LDL-C measurements in AoU.
