# Supplementary material for: Precision Medicine in Cardiovascular Disease Prevention: Clinical Validation of Multi-Ancestry Polygenic Risk Scores in a U.S. Cohort
Source: Nutrients. 2025 Mar 6;17(5):926. doi: 10.3390/nu17050926 (PMC11901995; doi:10.3390/nu17050926)
Supplement: Supplementary file 1 [file nutrients-17-00926-s001.zip › nutrients-3478718-supplementary.pdf]

Supplementary Material

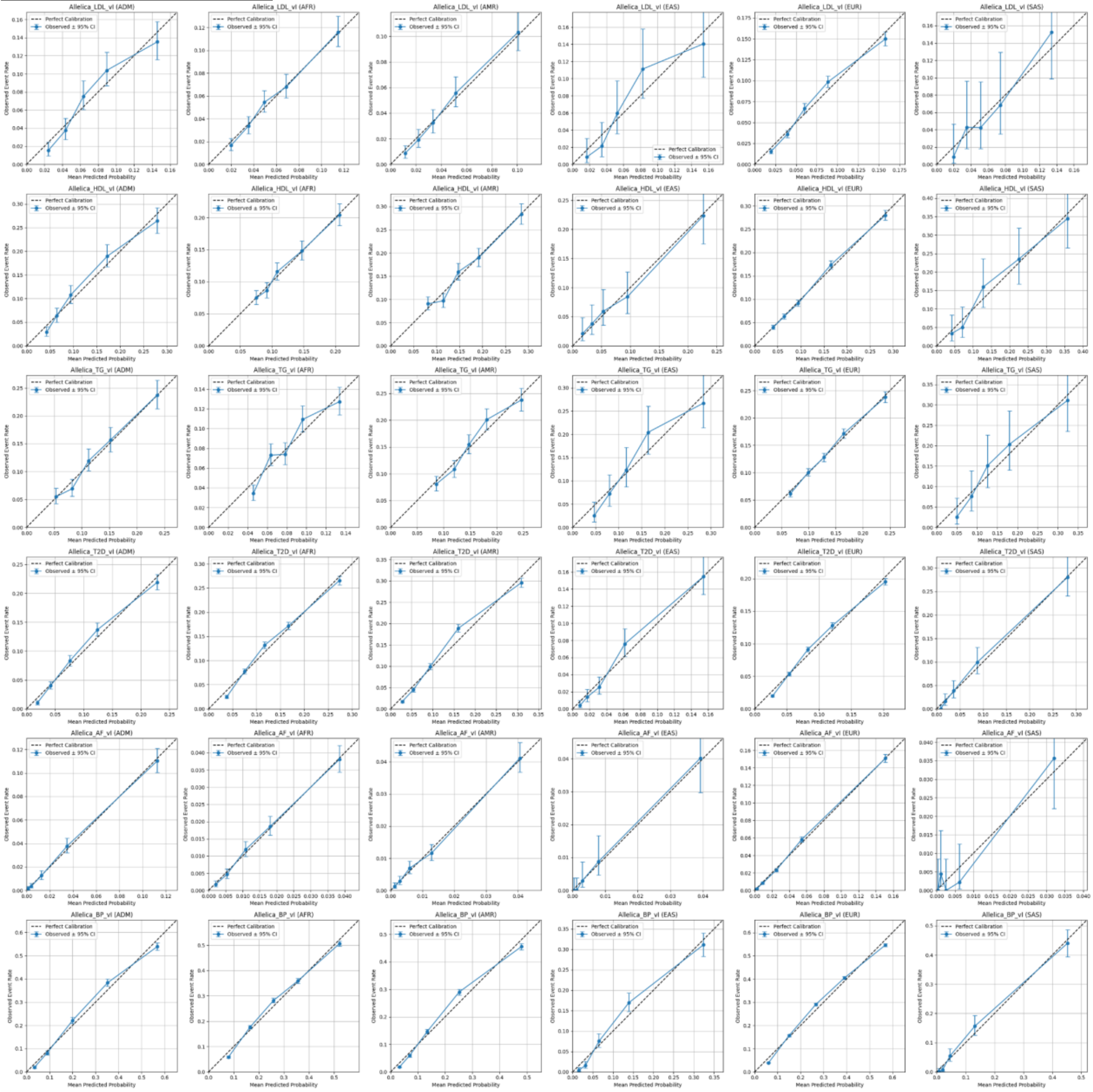

Figure S1. Cardiometabolic traits (AF, HT, T2DM) and lipid-related traits (LDL >160 mg/dL, HDL, and triglycerides) assessed for calibration performance across ancestry specifics predictive models

Table S1. Concept IDs and corresponding descriptions used to identify LDL-C measurements in AoU.

Concept ID	Concept Description
1175998	Cholesterol in LDL 2 [Moles/volume] in Serum or Plasma
3001308	Cholesterol in LDL [Moles/volume] in Serum or Plasma
1176351	Cholesterol in LDL 2 [Mass/volume] in Serum or Plasma
36031404	Cholesterol in LDL [Mass/volume] in Serum or Plasma by Calculated by Martin-Hopkins
3028437	Cholesterol in LDL [Mass/volume] in Serum or Plasma
3053190	Cholesterol in LDL real size pattern [Identifier] in Serum or Plasma
1175279	Cholesterol in LDL 5 [Mass/volume] in Serum or Plasma
3053341	Cholesterol in LDL [Mass/volume] in Serum or Plasma by Electrophoresis
42870529	Cholesterol in LDL [Moles/volume] in Serum or Plasma by Direct assay
1175794	Cholesterol in LDL 7 [Moles/volume] in Serum or Plasma
3038988	Cholesterol in LDL [Moles/volume] in Serum or Plasma by calculation
3009966	Cholesterol in LDL [Mass/volume] in Serum or Plasma by Direct assay
3030437	Cholesterol in LDL.narrow density [Mass/volume] in Serum or Plasma
3028288	Cholesterol in LDL [Mass/volume] in Serum or Plasma by calculation
36031267	Cholesterol in LDL [Moles/volume] in Serum or Plasma by Calculated by Martin-Hopkins
3045323	Cholesterol in LDL acylated [Mass/volume] in Serum or Plasma
3035899	Cholesterol in LDL [Mass/volume] in Serum or Plasma ultracentrifugate
1175959	Cholesterol in LDL 1 [Mass/volume] in Serum or Plasma
1176192	Cholesterol in LDL 7 [Mass/volume] in Serum or Plasma
3035009	Cholesterol in LDL [Units/volume] in Serum or Plasma by Electrophoresis
3050730	Cholesterol in LDL pattern BI [Presence] in Serum or Plasma by Electrophoresis
1175225	Cholesterol in LDL 6 [Moles/volume] in Serum or Plasma
1175381	Cholesterol in LDL 6 [Mass/volume] in Serum or Plasma
3008631	Cholesterol in LDL [Percentile]
3033200	Cholesterol in LDL [Mass or Moles/volume] in Serum or Plasma
1175948	Cholesterol in LDL 4 [Moles/volume] in Serum or Plasma
36032416	Cholesterol in LDL [Mass/volume] in DBS by Direct assay
3039873	Cholesterol in LDL [Moles/volume] in Body fluid
3049237	Cholesterol in LDL pattern A [Presence] in Serum or Plasma
3052982	Cholesterol in LDL pattern BII [Presence] in Serum or Plasma by Electrophoresis
1175571	Cholesterol in LDL 3 [Moles/volume] in Serum or Plasma
1175729	Cholesterol in LDL 3 [Mass/volume] in Serum or Plasma
1175617	Cholesterol in LDL 5 [Moles/volume] in Serum or Plasma

Concept ID	Concept Description
1175889	Cholesterol in LDL 1 [Moles/volume] in Serum or Plasma
1175898	Cholesterol in LDL 4 [Mass/volume] in Serum or Plasma

Table S2. Concept IDs and corresponding descriptions used to identify HDL-C measurements in AoU.

Concept ID	Concept Description
3034482	Cholesterol in HDL [Units/volume] in Serum or Plasma by Electrophoresis
3033011	Cholesterol in HDL 2+3 [Mass/volume] in Serum or Plasma
3023574	Cholesterol in HDL 3 [Mass/volume] in Serum or Plasma
40759254	Cholesterol in HDL 2b [Moles/volume] in Serum or Plasma
3001318	Cholesterol.total/Cholesterol in HDL [Percentile]
40757503	Cholesterol in HDL [Mass/volume] in Body fluid
36032229	Cholesterol in HDL [Mass/volume] in DBS
3020107	Cholesterol in HDL 2 [Mass/volume] in Serum or Plasma ultracentrifugate
40757602	Cholesterol in HDL 3c/Cholesterol in total HDL in Serum or Plasma by Electrophoresis
3050988	Cholesterol in HDL 4+5 [Mass/volume] in Serum or Plasma
40757601	Cholesterol in HDL 3b/Cholesterol in total HDL in Serum or Plasma by Electrophoresis
3053286	Cholesterol in HDL [Mass/volume] in Serum or Plasma by Electrophoresis
3007070	Cholesterol in HDL [Mass/volume] in Serum or Plasma
3020189	Cholesterol in HDL 2 [Moles/volume] in Serum or Plasma
40757600	Cholesterol in HDL 3a/Cholesterol in total HDL in Serum or Plasma by Electrophoresis
40757598	Cholesterol in HDL 2a/Cholesterol in total HDL in Serum or Plasma by Electrophoresis
3005984	Deprecated Cholesterol.in HDL [Mass/volume] in Serum or Plasma
3009718	Cholesterol in HDL 2 [Mass/volume] in Serum or Plasma
3005561	Cholesterol in HDL 3 [Moles/volume] in Serum or Plasma
40759253	Cholesterol in HDL 2a [Moles/volume] in Serum or Plasma
3040815	Cholesterol in HDL [Moles/volume] in Dialysis fluid
3033190	Cholesterol in HDL [Mass or Moles/volume] in Serum or Plasma
3032633	Cholesterol in HDL 3 [Mass or Moles/volume] in Serum or Plasma
40761043	Cholesterol in HDL 3a [Mass/volume] in Serum or Plasma
3023602	Cholesterol in HDL [Moles/volume] in Serum or Plasma
40757599	Cholesterol in HDL 2b/Cholesterol in total HDL in Serum or Plasma by Electrophoresis

Concept ID	Concept Description
3011884	Cholesterol in HDL [Presence] in Serum or Plasma
3030792	Cholesterol in HDL [Moles/volume] in Body fluid
3033638	Cholesterol in HDL [Presence] in Serum or Plasma by Electrophoresis
40761042	Cholesterol in HDL 2a [Mass/volume] in Serum or Plasma
3013473	Cholesterol in HDL [Mass/volume] in Serum or Plasma ultracentrifugate
3050038	Cholesterol in HDL 1+2 [Mass/volume] in Serum or Plasma

Table S3. Concept IDs and corresponding descriptions used to identify Triglycerides measurements in AoU.

Concept ID	Concept Description
3035817	Triglyceride in Lipoprotein a [Mass/volume] in Serum or Plasma
4041722	Plasma fasting triglyceride measurement
4055696	Plasma random triglyceride measurement
4055695	Plasma triglyceride measurement
3466880	Plasma random triglyceride measurement
4042590	Serum fasting triglyceride measurement
4055667	Serum random triglyceride measurement
3447812	Serum fasting triglyceride measurement
3026876	Triglyceride+ester in LDL [Mass/volume] in Serum or Plasma
40308383	Plasma fasting triglyceride level
3438724	Measurement of serum triglyceride level
3026041	Triglyceride+ester in IDL [Mass/volume] in Serum or Plasma
3033960	Triglyceride in Lipoprotein a [Presence] in Serum or Plasma
3040324	Triglyceride in HDL 2 [Moles/volume] in Serum or Plasma
3457712	Serum random triglyceride measurement
4021289	Triglyceride and ester in HDL measurement
3048773	Triglyceride [Moles/volume] in Serum or Plasma --fasting
3557356	Serum triglycerides NOS
4020697	Triglyceride and ester in IDL measurement
4032789	Triglycerides measurement
4156816	Measurement of serum triglyceride level
3022038	Triglyceride [Mass/volume] in Blood
3009192	Triglyceride [Mass/volume] in Semen
3426917	Triglyceride and ester in intermediate density lipoprotein measurement
3456353	Triglyceride and ester in low density lipoprotein measurement

Concept ID	Concept Description
4017787	Lipids, triglycerides measurement
37394096	Serum random triglyceride level
3034111	Deprecated Triglyceride [Mass/volume] in Serum or Plasma --post CFst
37394233	Plasma random triglyceride level
3468622	Lipids, triglycerides measurement
3013678	Triglyceride [Mass/volume] in Serum or Plasma --12 hours fasting
4020119	Triglyceride and ester in LDL measurement
3444545	Plasma triglyceride measurement
3046441	Lipoprotein.pre-beta/Triglyceride [Mass Ratio] in Serum or Plasma
3019038	Triglyceride [Moles/volume] in Serum or Plasma --12 hours fasting
37208864	Triglyceride substance concentration in plasma
40307758	Serum random triglyceride level
3439725	Triglyceride and ester in high density lipoprotein measurement
3022192	Triglyceride [Mass/volume] in Serum or Plasma
37394386	Plasma fasting triglyceride level
3027997	Triglyceride [Mass/volume] in Serum or Plasma --fasting
36032380	Triglyceride [Mass/volume] in DBS
4353850	High density lipoprotein/triglyceride ratio measurement
37392563	Serum triglycerides level
3572267	Triglyceride level
3450648	Plasma fasting triglyceride measurement
40308380	Plasma triglyceride level
40307760	Serum triglycerides NOS
36660413	Triglyceride [Mass/volume] corrected for glycerol in Serum
3030875	Triglyceride [Mass or Moles/volume] in Serum or Plasma
4042591	Serum triglycerides NOS
3007943	Triglyceride [Mass/volume] in Serum or Plasma by calculation
37398563	Plasma triglyceride level
3552985	Triglyceride level
3012391	Triglyceride [Presence] in Serum or Plasma
3025202	Triglyceride+ester in HDL [Mass/volume] in Serum or Plasma
42868692	Triglyceride [Moles/volume] in Blood
40308382	Plasma random triglyceride level

Concept ID	Concept Description
37208865	Triglyceride substance concentration in serum
40307753	Serum triglycerides (& level)
40329067	Serum triglycerides (& level)
3014600	Triglyceride [Percentile]
3025839	Triglyceride [Moles/volume] in Serum or Plasma
44808857	Triglyceride level
40307757	Serum fasting triglyceride level
36660269	Glycerol and glycerol-corrected triglyceride panel - Serum or Plasma
3468184	High density lipoprotein/triglyceride ratio measurement
3050630	Triglyceride in HDL 3 [Moles/volume] in Serum or Plasma
3013898	Deprecated Triglyceride [Mass/volume] in Serum or Plasma
37394095	Serum fasting triglyceride level

Table S4. Drug names used to Identify Lipid Measurements for Potential Exclusion. This table lists the lipid-lowering medications identified in the dataset, which were used to determine whether lipid measurements occurred after treatment initiation. Measurements taken post-treatment were excluded from the subsequent analyses.

fibrate	fibrate statin	niacin	niacin statin	statin	statin_cai	acli	sequestrant bile acid	cai and cai acli	pcsk9_inh
bezafibrate	fenofibrate-pravastatin	Endur-acin	Advicor	amolodipine-atorvastatin	ezetimibe-atorvastatin		Prevalite	Nexlizet	Praluent
Bezalip	Pravafenix	niacin	lovastatin-niacin	Caduet	Liptruzet	Nexletol	cholestyramine	Nustendi	alirocumab
Atromid-s	Cholib	Niacin SR	Simcor	Atorvaliq	ezetimibe-rosuvastatin	Nilemdo	Questran	bempedoic acid-ezetimibe	Repatha
clofibrate	fenofibrate-simvastatin	Niacin-50	simvastatin-niacin	atorvastatin	Roszet	bempedoic	Welchol	Zetia	evolocumab
Antara		Niacor		Lipitor	ezetimibe-simvastatin		Cholestagel	ezetimibe	
Atorva TG		Slo-Niacin		Baycol	Vytorin		colesevelam		
Fenocor		Endur-Amide		cerivastatin			Lodalis		
fenofibrate		niacinamide		Lipobay			Colestid		
Fenogal		Niaspan		fluvastatin			colestipol		
Fenoglide		nicotinamide		Lescol			Cholestabyl		
Fibricor		nicotinamide		Altocor			Lestid		

fibrate	fibrate statin	niacin	niacin statin	statin	statin_cai	acli	sequestrant bile acid	cai and cai acli	pcsk9_inh
		adenine dinucleotide							
Golip		nicotinamide riboside		Altoprev					
Lipanthyl		Tru Niagen		lovastatin					
Lipantil		nicotinic acid		Mevacor					
Lipidil		vitamin B3		Livalo					
Lipofen				pitavastatin					
Lofibra				Zypitamag					
Phenofibrate				Pravachol					
Procetofen				pravastatin					
Supralip				pravastatin-aspirin					
Tricheck				Pravigard					
Tricor				Crestor					
Triglide				Ezallor					
fenofibric acid				rosuvastatin					
Trilipix				FloLipid					
fibric acid				simvastatin					
gemfibrozil				Zocor					
Lopid				Juvisync					
				sitagliptin-simvastatin					

Table S5. Diagnoses, procedures, and CPT codes used to identify CAD outcomes in All of Us.

Diagnoses IDC9	Diagnoses IDC10	ICD9 procedures	CPT codes
410	I20.0	36.01-36.3	33140
412	I21	36.05	33533-33536
429.7	I22	36.09-36.19	33510-33523
411	I23		33530
413	I24.0		92920-92921
414	I24.8		92924-92925
V45.81	I24.9		92928-92929
V45.82	I25.2		92933-92934

Diagnoses IDC9	Diagnoses IDC10	ICD9 procedures	CPT codes
	I25.10		92937-92938
	I25.11		92941
	I25.7		92943-92944
	Z95.1		92980-92982
	Z95.5		92984
			92995-92996
			92974

Table S6. Concept IDs and corresponding descriptions used to identify AF outcomes in AoU.

Concept ID	Concept Description
45768480	Longstanding persistent atrial fibrillation
4154290	Paroxysmal atrial fibrillation
313217	Atrial fibrillation
4117112	Controlled atrial fibrillation
4232697	Persistent atrial fibrillation
4232691	Permanent atrial fibrillation
44782442	Atrial fibrillation with rapid ventricular res...
4064452	ECG: atrial fibrillation
4141360	Chronic atrial fibrillation
4199501	Rapid atrial fibrillation
4108832	Atrial fibrillation and flutter

Table S7. Concept IDs and Corresponding descriptions used to identify HT outcomes in AoU

Concept ID	Concept Description
44834715	Benign essential hypertension
35207668	Essential (primary) hypertension
44821949	Unspecified essential hypertension
44833556	Essential hypertension
44823109	Malignant essential hypertension

Table S8. Concept IDs and Corresponding descriptions used to identify T2DM outcomes in AoU

Concept ID	Concept Description
45757474	Diabetes mellitus type 2 without retinopathy

Concept ID	Concept Description
4099651	Type 2 diabetes mellitus with ulcer
201826	Type 2 diabetes mellitus
4130162	Insulin treated type 2 diabetes mellitus
4063043	Pre-existing type 2 diabetes mellitus
4304377	Type 2 diabetes mellitus in obese
4193704	Type 2 diabetes mellitus without complication
45766052	Type II diabetes mellitus in remission
4230254	Type 2 diabetes mellitus in nonobese
